# T-2 Toxin—The Most Toxic Trichothecene Mycotoxin: Metabolism, Toxicity, and Decontamination Strategies

**DOI:** 10.3390/molecules26226868

**Published:** 2021-11-14

**Authors:** Edyta Janik, Marcin Niemcewicz, Marcin Podogrocki, Michal Ceremuga, Maksymilian Stela, Michal Bijak

**Affiliations:** 1 Biohazard Prevention Centre, Faculty of Biology and Environmental Protection, University of Lodz, Pomorska 141/143, 90-236 Lodz, Poland; edyta.janik@edu.uni.lodz.pl (E.J.); marcin.niemcewicz@biol.uni.lodz.pl (M.N.); marcin.podogrocki@biol.uni.lodz.pl (M.P.); 2 Military Institute of Armament Technology, Prymasa Stefana Wyszyńskiego 7, 05-220 Zielonka, Poland; ceremugam@witu.mil.pl; 3 CBRN Reconnaissance and Decontamination Department, Military Institute of Chemistry and Radiometry, Antoniego Chrusciela "Montera" 105, 00-910 Warsaw, Poland; m.stela@wichir.waw.pl

**Keywords:** trichothecenes, type A, T-2 toxin, toxicity, metabolism, decontamination

## Abstract

Among trichothecenes, T-2 toxin is the most toxic fungal secondary metabolite produced by different *Fusarium* species. Moreover, T-2 is the most common cause of poisoning that results from the consumption of contaminated cereal-based food and feed reported among humans and animals. The food and feed most contaminated with T-2 toxin is made from wheat, barley, rye, oats, and maize. After exposition or ingestion, T-2 is immediately absorbed from the alimentary tract or through the respiratory mucosal membranes and transported to the liver as a primary organ responsible for toxin's metabolism. Depending on the age, way of exposure, and dosage, intoxication manifests by vomiting, feed refusal, stomach necrosis, and skin irritation, which is rarely observed in case of mycotoxins intoxication. In order to eliminate T-2 toxin, various decontamination techniques have been found to mitigate the concentration of T-2 toxin in agricultural commodities. However, it is believed that 100% degradation of this toxin could be not possible. In this review, T-2 toxin toxicity, metabolism, and decontamination strategies are presented and discussed.

## 1. Introduction

**Figure 1:**
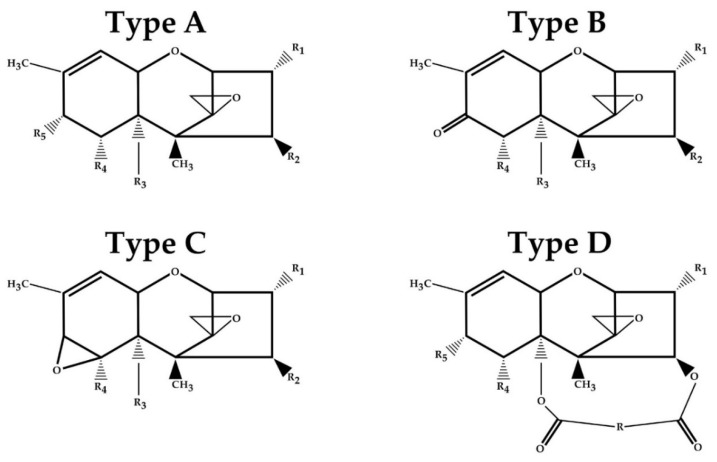
The core structures for A, B, C, and D trichothecenes (TCT) types.

Trichothecenes (TCT) are groups of chemically related mycotoxins compounds produced by diverse filamentous fungal species such as *Fusarium*, *Myrothecium*, *Stachybotrys*, *Trichoderma*, *Trichothecium*, and *Spicellum*, which pose a threat to human and animal health [1,2]. The fungi capable of producing TCT can be found throughout the world. They are able to grow under a variety of environmental conditions including the nutrient content, temperature, moisture content, and oxygen level in growth medium, which resulted in successful colonization [3,4]. TCT are non-volatile, low molecular weight (typically 200–500 Da) sesquiterpenoids synthesized by the terpenoid biosynthetic pathway [5,6]. They are slightly soluble in water but highly soluble in polar organic solvents such as ethyl acetate, chloroform, ethanol, methanol, and propylene glycol [7]. The TCT common structure consists of a three-ring molecule known as 12,13-epoxytrichothec-9-ene (EPT) (Figure 1) [8,9]. The cyclohexene (A-ring) is fused to the tetrahydropyran (B-ring), which is bridged by a two-carbon chain at C-2 and C-5, thus forming a cyclopentyl moiety (C-ring) [10]. TCT are divided based on the substitution pattern of EPT into four types (A–D) (Table 1) [11]. Type A TCT is distinguished by a hydroxyl (OH) group at C-8, an ester function at C-8, or no oxygen substitution at C-8 [12,13].

TCT are identified as a significant threat to human and animal health. The most toxic TCT include T-2, HT-2 toxin, deoxynivalenol (DON), nivalenol (NIV), and diacetoxyscirpenol (DAS) [14]. TCT toxicological activity is related to the presence of the epoxide at the C 12,13 position. Their mechanism of action is mainly based on the protein synthesis inhibition and oxidative cell damage, which is followed by the disruption of synthesis of nucleic acid and the resulting apoptosis [15]. The main sources of TCT in food and feed are wheat, barley, rye, oats, and maize [16,17,18]. They also occur in hay, straw, green feed, and silage from contaminated cereals [19]. What is more, *Myrothecium* species can contaminate various vegetables e.g., tomato [20]. TCT can also enter human food chains through breakfast cereals, bakery products, snack foods, and beer. Moreover, consumption of products such as meat, milk, and eggs from livestock and poultry that are fed with TCT-contaminated feed are the primary cause of human poisoning [21,22]. TCT are easily absorbed through the gastrointestinal membranes and distributed to different tissues and organs due to their low molecular weight and amphipathic nature. The consumption of TCT contaminated products may cause variable adverse effects including emesis, anorexia, carcinogenicity, hematotoxicity, immunotoxicity, and neurotoxicity [23,24,25,26]. Moreover, it has been reported that T-2 is extremely toxic to the mucous membranes and skin [27]. These effects depend on various factors including mycotoxin's toxicity, time of exposure, or individual nutritional status [28].

T-2 toxin, which belongs to the A TCT type, has the highest toxicity of all TCTs [14,29,30]. T-2 is produced by different *Fusarium* species, including *F*. *sporotrichioides*, *F*. *poae*, and *F*. *acuminatum* [31,32]. Their presence in a variety of cereal grains has been documented in cold and moderate climate regions and during wet storage conditions [33,34]. *F. sporotrichioides*, as the main T-2 producer, grows in the temperature ranging from −2 to 35 °C, preferably with water activity (aw) above 0.88 [35]. The temperature and aw optimal for the T-2 biosynthesis are 20–30 °C and aw in the range of 0.980–0.995, respectively [36]. T-2 is resistant to degradation in different environmental conditions, such as high temperature and UV light. However, it is effectively deactivated in a strong acid or alkaline environment, and it can be affected by the presence of coexisting fungi or bacteria leading to detoxifying T-2 by altering its chemical structure [29].

T-2 poses a potential threat to humans and animals as a natural cereals contaminant and can induce a wide range of toxic effects due to its strong toxicity. T-2 has different toxic effects depending on the dosage, age, and ways of exposure (oral, dermal, and aerosol). Generally, observed acute toxicological effects are feed refusal, vomiting, hemorrhages, stomach necrosis, and dermatitis [37,38]. In addition, T-2 is considered to be a main cause of a gastrointestinal disorder called alimentary toxic aleukia disease (ATA) affecting in history soldiers in World War II and humans in certain world regions after eating molded food [39,40].

The aim of this study is to characterize the T-2 mycotoxin with special attention paid to the aspect of the multidirectional toxicity, metabolism, and decontamination strategies.

## 2. Structure and Physical and Chemical Properties of T-2 Toxin

**Figure 2:**
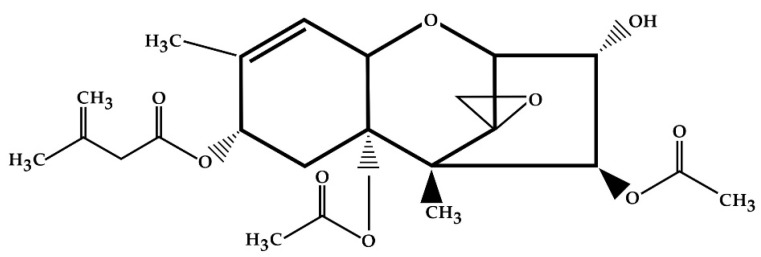
Chemical structure of T-2 toxin.

The T-2 toxin belongs to type A trichothecenes. As a TCT, it contains a double bond between C-9 and C-10 and an epoxy group between C-12 and C-13 [12]. The T-2 chemical structure is characterized by a hydroxyl (OH) group at the C-3 position, acetyloxy (-OCOCH_3_) groups at the C-4 and C-15 positions, an atom of hydrogen at the C-7 position and an ester-linked isovaleryl [OCOCH_2_CH(CH_3_)_2_] group at the C-8 position (Figure 2) [41].

## 3. Metabolism of T-2 Toxin

T-2 toxin has a lipophilic character and can be immediately absorbed from the alimentary tract or through the respiratory mucosal membranes [42]. Liver is the primary organ of toxin’s metabolism after its absorption [43]. After ingestion, toxin is rapidly absorbed and excreted in feces and urine. The half-life of T-2 in plasma is short, and elimination is usually completed within 48 h, depending on the administration mode, the consumed amount, and on species-specific differences [44]. In addition, toxin does not accumulate in significant quantity in various organs such as the kidneys, liver, or the skeletal muscle [45,46]. The major metabolic pathways are usually hydrolysis, hydroxylation, conjugation, and de-epoxidation [45]. Typical metabolites of T-2 toxin are HT-2 toxin, T-2-triol, T-2-tetraol, neosolaniol (NEO), 3′-hydroxy-T-2 (3′-OH-T-2), 3′-hydroxyHT-2 toxin (3′-OH-HT-2), deepoxy-3′-hydroxy-T-2-triol, deepoxy-3′-hydroxy-HT-2, 3′-hydroxy-T-2-triol, dihydroxy-HT-2 toxin, 3′7-dihydroxy-T-2 (3′,7′-di-OH-T-2), and 3′,7-dihydroxy-HT-2 toxin (3′,7′-di-OH-HT-2) [29]. In recent years, some in vitro and in vivo studies of T-2 bioconversion have been conducted. The performed studies have led to characterization of the metabolic pathways and identification of the main T-2 metabolites in different species.

Yang et al. [47] performed in vitro study with animal and human liver microsomes that aimed to investigate the phase I and II metabolites. In this study, T-2 was incubated with chickens, swine, goats, cows, rats, or humans liver microsomes under identical experimental conditions. As a consequence, four phase I metabolites (HT-2, NEO, 3′-OH-T-2, and 3′-OH-HT-2) and three phase II glucuronide binding metabolites (T-2-3-glucuronide (T-2-3-GlcA), HT-2-3-glucuronide (HT-2-3-GlcA), HT-2-4-glucuronide (HT-2-4-GlcA)) of T-2 were discovered. The HT-2 toxin was the predominant metabolite in all species, suggesting that the HT-2 may serve as a biomarker allowing to assess the dietary exposure of animals and humans to T-2. The T-2 possible metabolic pathways mainly consist of hydrolysis (HT-2, NEO), hydroxylation (3′-OH-T-2 and 3′-OH-HT-2), and glucuronidation (T-2-3-GlcA, HT-2-3-GlcA, HT-2-4-GlcA). However, a significant metabolic difference was observed among species. Compared to other in vitro models, a large amount of unmetabolized T-2 remained after incubation with chicken liver microsomes, especially in the phase II incubation system. It suggests that the chickens have a lower ability to metabolize and conjugate T-2. Moreover, a significant difference was observed on the hydroxylated products. 3′-OH-T-2, 3 was the main hydroxylated product observed in chickens, cows, and rats, while for goats, swine, and humans, it was 3′-OH-HT-2. In addition, species-specific patterns of T-2 glucuronidation were also noticed. The major glucuronidation product in cows and goats was T-2-3-GlcA, while for the other animal species and human, it was HT-2-3-GlcA [47]. In vitro studies with rat liver microsomes and liver S9 fraction were used. The results showed that hydrolysis was the main metabolic pathway of T-2 toxin, followed by hydroxylation. The HT-2, NEO, 9′-hydroxy-T-2 (9-OH-T-2), and 4-deacetylneosolaniol were the main metabolites in liver microsomes systems, whereas HT-2, 4-deacetylneosolaniol (4-deAc-NEO), NEO, 9-OH-T-2, and 3′-OH-T-2 had high contents in liver S9 fraction systems [43].

An in vivo study was performed by Yang and colleagues [47], which aimed to investigate the metabolism of T-2 in chickens after oral administration. As a result, 18 metabolites (Table 2) were detected and identified in the chickens bile and feces. Some of these metabolites such as 3′-Hydroxy-T-2-3-sulfate (3′-OH-T-2 3-SO3H), 3′-Hydroxy-HT-2-3-sulfate (3′-OH-HT-2 3-SO3H), 4′-Hydroxy-HT-2 (4′-OH-HT-2), 3′,4′-Dihydroxy-T-2 (3′,4′-di-OH-T-2), 4′-Carboxyl-T-2 (4′-COOH-T-2), 4′-Carboxyl-HT-2 (4′-COOH-HT-2), 4′-Carboxyl-3′-hydroxy-T-2 (4′-COOH-3′-OH-T-2), and their isomers were discovered. T-2 was extensively metabolized in chickens demonstrated by the recovery of only traces of unmetabolized toxin in chicken excreta. This study showed that 3′-OH-HT-2 was the main metabolite of T-2 [47].

What is more, the same results were obtained in a study with rats [43]. These results suggested that in rats and chickens, T-2 was hydrolyzed to HT-2, and it could undergo hydroxylation at the isovaleryl group and produce 3′-OH-HT-2. Therefore, this metabolite may serve as a T-2 biomarker of exposure. What is more, two novel metabolites (3′-OH-T-2 3-SO3H, 3′-OH-HT-2 3-SO3H) indicate that the sulfonation may be a T-2 specific metabolic pathway in chickens [47].

In vivo studies in rats as an animal model revealed a significant difference between male and female rats concerning the type of T-2 toxin metabolites. For male rats, the main metabolite of T-2 toxin was 3′-OH-HT-2 followed by de-epoxy-3′-OH-HT-2, 3′,7′-di-OH-T-2, HT-2, 3′-OH-T-2, 4-deAc-NEO, and 7′-hydroxy-HT-2 (7′-OH-HT-2). In comparison, for the female rats, the main metabolites were HT-2, 3′-OH-HT-2, de-epoxy-3′-OH-HT-2, 3′-OH-T-2, 9-OH-T-2, and 4-deAc-NEO, sequentially [43].

## 4. T-2 Toxicity

**Figure 3:**
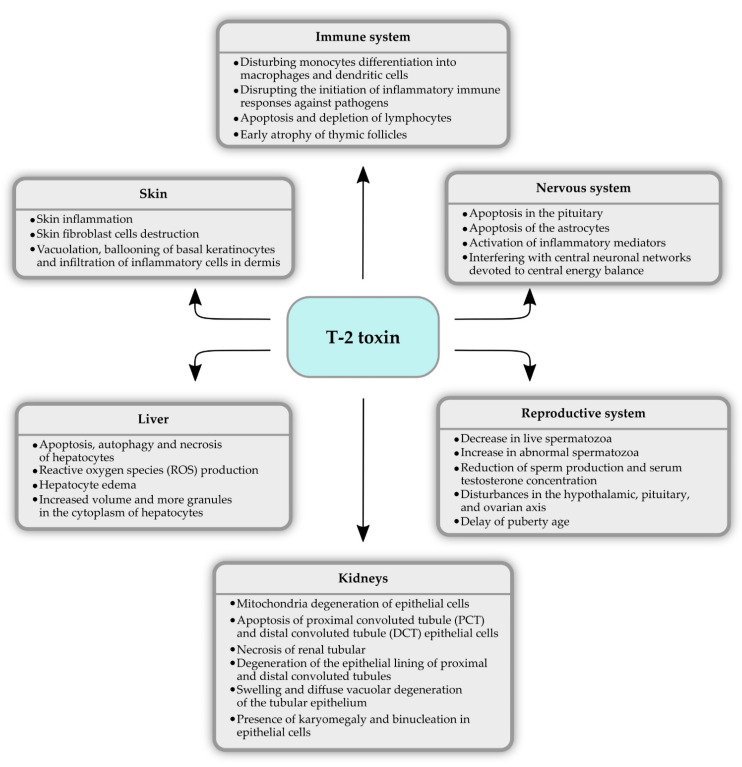
The main toxic effects of T-2 toxin in the organism.

Many studies have been performed in the last few decades focusing on the cytotoxic and genotoxic effects of T-2 toxin. At a cellular level, the major effect of T-2 is inhibition of protein synthesis, which leads to secondary DNA disruption and RNA synthesis [48]. T-2 is hypothesized to bind and inactivate peptidyl-transferase activity at the transcription site, resulting in the inhibition of protein synthesis. The most important molecular target of TCT is the 60S ribosomal unit, where it prevents polypeptide chain initiation. This inhibitory effect is most visible in actively proliferating cells such as the gastrointestinal tract, skin, thyroid, bone marrow, and erythroid cells [49,50]. The oxidative stress associated with detrimental effects, such as elevated lipid peroxidation, nuclear and mitochondrial DNA damage, disturbances in the cell signaling, and inflammatory pathways are also the effects of T-2 toxin intoxication. What is more, toxin affects the cell cycle and induces apoptosis [51,52,53]. Both in vitro and in vivo studies confirmed the toxic properties of this mycotoxin (Figure 3), and the results of some of them are presented below.

### 4.1. Hepatotoxicity

Ihara and colleagues [54] investigated whether T-2 possesses an ability to induce apoptosis in a mice model. The analysis revealed that the DNA fragmentation in liver happened shortly after exposition to the toxin. The induction of apoptotic cellular lesions and phagocytosis of apoptotic bodies by Kupffer cells was observed 2 hours after toxin administration. These lesions were not observed 12 hours after receiving T-2 [54]. In an in vivo study, Yin et al. [55] assessed the toxicological effect of T-2 on apoptosis and autophagy in chicken hepatocytes. The apoptosis rate and pathological changes degree hepatocytes increased in a dose-dependent manner. Histopathological analysis showed that the toxin caused pathological changes in liver tissue, including hepatocyte edema, increased volume, and more granules in the cytoplasm. It suggests that the exposition to the T-2 leads to hepatocyte apoptosis. At the molecular level, T-2-induced mitochondria-mediated apoptosis was caused by producing reactive oxygen species (ROS) and promoting cytochrome c (cyt c) translocation between mitochondria and cytoplasm. What is more, the expression of the autophagy-related proteins such as Beclin-1, ATG5, and ATG7 and the LC3-II/LC3-I ratio were increased. It suggests that T-2 caused autophagy. Further experiments showed that the phosphoinositide 3-kinase (PI3K)/protein kinase B (AKT)/mammalian target of rapamycin (mTOR) signal may be involved in autophagy induced by T-2 in chicken hepatocytes [55]. An in vivo study with mice revealed the up-regulated expression of oxidative stress and apoptosis-related genes and the down-regulated expression of glycogen metabolism-, lipid metabolism-, drug metabolism- and blood coagulation-related genes. In particular, c-fos and c-jun expression was notably elevated immediately after T-2 toxin administration and remained at a high level up to 24 hours after. Moreover, T-2 induced death in a small number of hepatocytes 3 hours after administration, and dead hepatocytes at the early stage corresponded to necrosis, while at the late stage they corresponded to apoptosis, respectively [56].

### 4.2. Nephrotoxicity

A toxicopathological study of T-2 effects on the kidneys in juvenile goats was conducted. The histological analysis revealed the changes in the kidneys after 30 days of T-2 toxin-contaminated diet. The nucleus and mitochondria showed extensive degeneration, and the mitochondria were the most affected organelles. Affected epithelial cells had a loss of cristae, leading to the creation of empty space and rendering the mitochondria to pleomorphic forms (variable sizes and shapes—rounded, dumb bell, curved). Heterochromatin condensation and margination with an indistinct nuclear membrane were also noticed. Within the kidney tissues, proximal convoluted tubule (PCT) and distal convoluted tubule (DCT) epithelial cells exhibited apoptotic changes. In general, the findings showed dose and duration-dependent modifications. Pathomorphological alterations included interstitial engorgement, degeneration of the epithelial lining of proximal and distal convoluted tubules, and renal tubular necrosis. All of these alterations in the renal tissues indicate the toxin’s harmful effect on kidneys [14]. A similar study with rats also showed that T-2 induced nephrotoxicity. Biochemical analysis showed increased levels of blood urea nitrogen (BUN) and serum creatinine. A significant increase in oxidative stress enzymes such as malondialdehyde (MDA) and decrease in superoxide dismutase (SOD), catalase, and glutathione (GSH) in kidneys enhanced the role of free radicals in causing kidney damage. The main renal histological changes were the swelling and diffuse vacuolar degeneration of the tubular epithelium. After 12 weeks of toxin-contaminated diet, almost all animals showed severe degenerated PCT epithelial cells, obliterating the lumen with the presence of denuded cells and protein aceous material in their lumina. What is more, the presence of karyomegaly and binucleation in epithelial cells was observed. The mononuclear cell infiltration around glomeruli and in the interstitium was also recorded in rats [57].

### 4.3. Immunotoxicity

Minervini et al. [58] conducted an in vitro study to investigate T-2 immunotoxicity effects on two lymphoid human cell lines, MOLT-4 (T lineage) and IM-9 (B lineage). As a result, cytotoxicity appeared to be due to early apoptosis in MOLT-4 cells, as indicated by the activation of caspase-3, and to direct cell membrane damage in IM-9 cells. Reduced viability (58%) was observed on the IM-9 line after 8 h of toxin administration. MOLT-4 showed a membrane damage (41% of cell viability) only after 24 h incubation at the higher than IM-9 line toxin concentration [58]. In a different in vitro study [59], the effect of T-2 toxin on human monocyte differentiation into macrophages and dendritic cells was shown. According to the results, T-2 is cytotoxic on monocytes during the differentiation process (in dendritic cells or macrophages, the results are similar). After 24 h of incubation, only 32% of cells survived after 24 of incubation. What is more, 2% of immature dendritic cells and 9% of macrophages were viable after 24 hours of incubation with toxin. CD71 (specific phenotypic macrophages cells marker) expression was downregulated to 40% after 6 days of culture in the condition, inducing monocyte differentiation into macrophages in the presence of toxin, whereas 91% of cells cultured without toxin expressed CD71. In addition, the expression of CD1a (specific dendritic cells marker) was downregulated, while the CD14 (specific monocyte marker) was upregulated. These results showed that the T-2 disturbs the human monocytes’ differentiation process into macrophages and dendritic cells. In another study [60], the evaluation of the effects of T-2 on the activation of macrophages by various agonists of Toll-like receptors (TLR) using an in vitro model of primary porcine alveolar macrophages (PAM) was performed. It was established that the ingestion of low concentrations of T-2 can alter TLR activation by decreasing the pattern recognition of pathogens, thereby disrupting the initiation of inflammatory immune responses against viruses and bacteria. Based on this finding, it could be hypothesized that the exposure to low concentrations of T-2 can increase the animals’ and humans’ susceptibility to opportunistic infections.

Rahman and colleagues [57] showed the immunopathological effects of T-2 mycotoxicosis in rats. T-2 toxicity caused the suppression of both humoral and cellular immune responses in a dose and duration-dependent manner. Suppression was followed by decreased serum immunoglobulin G (IgG), immunoglobulin M (IgM), immunoglobulin A (IgA) levels, hemagglutination (HA) titers, delayed type hypersensitivity (DTH) response to ovalbumin, CD4+:CD8+ ratios, and the number of CD4+ and CD8+ lymphocytes in peripheral blood and mRNA expression levels of cytokines such as interleukin 2 (IL-2), interferon gamma (IFN-γ), interleukin 4 (IL-4), and interleukin 10 (IL-10) in toxin-fed animals. In addition, lymphoid organs (spleen, thymus, and Peyer’s patches) changes were observed. Changes in all organs were of a similar nature but were more severe in thymus than in spleen and Peyer’s patches. The depletion of lymphocytes started as single-cell apoptosis, then forming large foci of lymphocytolysis, especially in the thymus. Changes in thymus also included inter and intrafollicular hemorrhage and increased interfollicular connective tissue, resulting in early atrophy of thymic follicles [57].

### 4.4. Neurotoxicity

Agrawal et al. [61] investigated the mechanism of T-2 toxin-induced apoptosis in a human neuroblastoma (IMR-32) cell line. A study showed that the apoptosis is induced through multiple signal transduction pathways. The exposition on IMR-32 cells to T-2 toxin is characterized by the generation of ROS and then loss of mitochondrial membrane permeability, caspase-3 activation, nuclear fragmentation, oligonucleosomal DNA fragmentation, poly (ADP-ribose) polymerase (PARP) cleavage, and apoptosis. Additionally, ROS can directly activate the Ras–Raf–MEK–extracellular signal-regulated kinase (ERK)–mitogen-activated protein kinase (MAPK) signal transduction pathway, leading to cell cycle arrest and apoptosis [61]. A different in vitro study showed that T-2 induces neurotoxicity in a mouse neuroblastoma2a (N2a) cell line in both a dose and time-dependent manner. A study revealed that toxin exposure inhibits the Nrf2/heme oxygenase-1 (HO-1) pathway and p53 activation, which leads to abnormal mitochondrial function and oxidative stress, which together contribute to caspase-dependent apoptotic cell death [62]. In another study, normal human astrocytes (NHA) in primary culture were used to investigate the apoptotic effects and accumulation of T-2 toxin. According to the results, human astrocytes were highly sensitive to the T-2 properties at low concentration. An increase in caspase-3-activity was reported 6 h after exposure to T-2, increasing up to 24 h. What is more, a strong accumulation of toxin was detected, and a study revealed a fast cellular uptake and high accumulation in NHA cells, leading to a 15- to 30-fold increased concentration in the intracellular compartment [63].

A study with rats showed pathological lesions in the brain three days after exposure to the T-2 toxin and damage in the pituitary seven days after exposure. Autophagy in the brain and apoptosis in the pituitary suggest that this toxin can induce various acute reactions in different tissues. Additionally, toxin was detected in the brain with low concentrations in rat, suggesting that T-2 may cross the blood–brain barrier (BBB). It is also possible that the detection of the toxin in the rat brain can be explained by individual differences in T-2 absorption and metabolism in different experimental animals [64]. Gaige et al. [65] provided that the T-2 toxin modifies feeding behavior by interfering with central neuronal networks devoted to central energy balance in a mice animal model. The results also suggest that inflammatory mediators partake in the toxin-induced anorexia and other symptoms such as reduced water intake, energy expenditure, body temperature, glycaemia, and locomotor activity. T-2 toxin ingestion resulted in the activation of several brain nuclei such as nucleus tractus solitarus (NTS), dorsal motor nucleus of the vagus (DMV), arcuate nucleus (Arc), paraventricular nucleus (PVN), and central amygdala (CeA) involved in the autonomic and endocrine regulation of feeding behavior and physiology. The authors suggest that cytokines from peripheral organs may signal the brain via neuronal and humoral pathways to modify animal homeostasis [65].

### 4.5. Reproductive System

An in vivo study that aimed to evaluate the toxic effect of T-2 on a reproductive system revealed that this toxin affects male mice fertility [66]. The results showed that the number of live spermatozoa decreased significantly. Moreover, the number of abnormal spermatozoa increased notably, and a remarkable decrease in spermatozoa with integrated acrosome was observed in mice treated with T-2 at both low and high doses. The efficiency of sperm production and serum testosterone concentration, testicular, and cauda epididymal sperm counts were significantly reduced in a dose-dependent manner. In addition, a low pregnancy rate and high fetal resorption rate were noticed when female mice were mated with toxin-exposed males. In a different study, Yang et al. [67] investigated spermatogenesis disorders in male mice caused by T-2 toxin exposition. Their studies also showed that the T-2 hinders the spermatogenesis, which is reflected in the decreased spermatozoa count and increased spermatozoa deformity rate. T-2 toxin exposition increased the ROS and MDA level and decreased the total anti-oxidation capacity (T-AOC) and the SOD activity in performed testes. In addition, an increased expression of caspase-3, caspase-8, caspase-9 mRNA, and Bax and inhibition of Bcl-2 expression were demonstrated. It suggests that spermatogenesis disorders caused by T-2 are related with germ cell apoptosis and mediated by oxidative stress [67]. The effects of maternal T-2 exposure (during gestation and lactation) on the development of testis in the mice offspring were investigated. The results showed significant decreases in body weight and testicular weight at puberty in male offspring. Toxin exposition led to the inhibition of an antioxidant system in testis by oxidative stress and decreased testosterone synthesis, and it also led to a decrease of testosterone levels at pre-puberty. What is more, a significant reduction in the gene expression levels of StAR and 3β-HSD that are involved in testosterone synthesis were noticed. Additionally, results revealed that maternal exposure to the toxin had no notable effects on the expression of genes related to apoptosis. In pre-puberty, the offspring of mice maternally exposed to T-2 tended to decrease the expression of apoptosis-related genes. However, maternal exposure to toxin had no significant impact on the offspring testis after sexual maturity, suggesting a return to reproductive function [68]. A similar study conducted by Perveen and colleagues [69] was performed. They investigated the effect of gestational and lactational exposure to the T-2 and its effects on the puberty of female mice offspring. The findings reported that postnatal exposure to the toxin delayed puberty age, which appears to be influenced by the stage of the estrus cycle. The results also showed that lactational exposure to the toxin induced disturbances in the hypothalamic, pituitary, and ovarian axis and caused oxidative damage. The mechanisms of the toxic effect of T-2 toxin on the reproduction system could be resulted by down-regulation of the mRNA level of hypothalamic Gnrh, pituitary Gnrhr, Lhb, and Fshb, and ovarian Lhr and Fshr, causing the interference with the relative expression of steroidogenesis genes and disrupting the synthesis of estrogen and progesterone [69]. In an in vitro study, the impact of T-2 on reproductive activity in pigs was investigated in porcine granulosa cell [70]. It was found that T-2 toxin has potent dose-dependent inhibitory effects on granulosa cell proliferation and steroidogenesis. The toxin strongly inhibited follicle-stimulating hormone (FSH) and insulin-like growth factor 1 (IGF-I) and induced progesterone production as well as granulosa cell proliferation.

### 4.6. Dermal Toxicity

Compared to other representatives of the trichothecenes, T-2 toxin has a toxic effect on the skin. Skin inflammation, skin fibroblast cells destruction, and skin damages similar to injuries caused by radiation are major topical effects of T-2 toxin [71]. The toxicity of T-2 in swine following topical application was investigated. The results showed that skin at the site of application was swollen and initially red and progressively turned dark red and purple. By day seven, at the edge of the exposed area, clefts were formed and were covered with serosanguinous exudate. These lesions were characterized as a sponge-like inflammation and progressed to locally extensive necrotizing dermatitis. After seven days, the skin was focally separated from the underlying tissue and covered with a thick scab. Morphological changes in the internal organs were minimal and were based on the necrosis of single cells in the follicles of lymphoid tissues and in the exocrine pancreas [72]. Agrawal et al. [73] investigated histological and biochemical alterations of inflammation and cutaneous injury caused by T-2 in mice. The histological changes included degenerative alterations such as vacuolation, ballooning of basal keratinocytes, and infiltration of inflammatory cells in dermis. Topical application of toxin resulted in skin oxidative stress in the form of increased ROS generation, lipid peroxidation, and neutrophil mediated myeloperoxidase activity. The analysis of matrix metalloproteinases (MMP)-9 and 2 showed MMP activation and their role in degenerative skin histological alterations. The results also revealed an increase in inflammatory cytokines, a significant increase in the levels of phosphorylated p38 MAPK, and an increase in the sub-G1 population at all toxin doses and time points indicating apoptosis. To summarize, T-2 induced skin injury was mediated by oxidative stress, MMP activity, the activation of myeloperoxidase, the activation of p38 MAPK and apoptosis of epidermal cells and consequently led to degenerative skin histological alterations [73].

## 5. T-2 Degradation and Mitigation Strategies

Integrated mycotoxin contamination preventive practices could minimize the presence of T-2 toxin in food. Operations including pre-harvest control (e.g., appropriate sowing dates, balanced fertilization, pest infestation management, and selection of resistant varieties), harvest control (e.g., proper timeliness of harvest, reduction of mechanical damages, effective cleaning), and post-harvest strategies (e.g., efficient drying and good storage practices) should mitigate mycotoxin production in agricultural commodities [74]. However, it may not be possible to completely prevent the formation of T-2 in agricultural products, and decontamination strategies involving physical, chemical, and biological techniques need to be used to decontaminate T-2 toxin [75].

### 5.1. Physical Methods

Segregation, cleaning, milling, boiling, roasting, irradiation, and microwave heating are reported as commonly used physical methods for various mycotoxin control [74]. However, because of T-2’s heat-stable nature, cooking processing such as boiling, baking, and extrusion cannot provide a 100% degradation rate of toxin from products [76]. The use of color sorting in order to remove the discolored oat groats can reduce the mycotoxin's level in end products of oat flake. The results showed that more than 90% of T-2 toxin can be removed during industrial processing [1]. According to De Angelis et al. [77], during bread-baking, T2 mitigation up to 74% was observed in naturally contaminated wheat flour. In another study [78], flaked oats were artificially contaminated and processed at the laboratory scale. During biscuit making, up to 45% of T-2 toxin was thermally degraded at 200 °C for 30 min. Different feed adsorbents were developed as an effective strategy to reduce mycotoxins. They have specific structures that allow them to absorb and trap target mycotoxins in feed. Several types of montmorillonite (MMT) clay were tested for their ability in binding T-2 in maize. Sodium montmorillonite (Na-MMT) was more effective than unmodified MMT because of the presence of Na+ ion, an alkali metal ion, which made the clay electrically neutral. As a consequence, the electrically neutral clay increased the binding of T-2 toxin. The Na-MMT is able to decontaminate 66% of T-2 in maize when applied at the level of 8%. Lemongrass powder mixed with MMT (LGP-MMT) was the second most efficient. LGP-MMT at 12% decontaminated 56% of toxin in maize. LGP-MMT contributed MMT clay that was more hydrophobic than the unmodified MMT. T-2 toxin, being a non-polar mycotoxin, attracts hydrophobic compounds and hence binds to the surface of the LGP-MMT [75]. Wei et al. [79] evaluated the ability of modified hydrated sodium calcium aluminosilicate (HSCAS) adsorbent to reduce the toxicity of T-2 in broilers. It was found that T-2 induced growth performance reduction, hepatic and small intestinal injuries in the group that was fed with a basal diet containing T-2 toxin. Dietary supplementation of the modified HSCAS adsorbent mitigated these injuries.

### 5.2. Chemical Methods

Some chemical agents have been indicated as decontaminants dedicated for mycotoxins. Chemicals used nowadays for the decontamination of mycotoxins could be divided into the following categories: alkaline (ammonia gas, sodium hydroxide, calcium hydroxide); acids (acetic acid, phosphoric acid, formic acid, propionic acid); reducing agents (sodium bisulfite, sugars: d-glucose or d-fructose); oxidizing agents (ozone, hydrogen peroxide); other (chlorinating agents, salts, and other miscellaneous reagents). All of them have efficiency related to particular mycotoxin [80]. However, there are not many publications related to efficiency versus T-2 toxin. There are data demonstrating that bases, oxidizing agents, and organic acids are suitable for A type trichothecenes decontamination, but they are insufficient for T-2 toxin decontamination [23,81].

The treatment of 0.25% NaClO in 0.25 M NaOH for four hours is able to inhibit the biological activity of T-2 toxin and other trichothecenes. NaClO has also been acclaimed as a decontamination agent for the T-2 toxin. However, these methods cannot be applicable for all food staff [50]. Typical food processing could also reduce mycotoxins concentration; for instance, water-soluble toxins could be washed out partially from the surface of grains. Washing barley and corn three times reduces DON (deoxynivalenol) content by 65 to 69%, while ZEN (zearalenone) reduces it by 2 to 61%. Since T-2 solubility in water is closer to ZEN (T-2 347 mg/L, ZEN 117 mg/L while DON—36,000 mg/L), a rather smaller reduction should be expected [23]. Reinholds et. al. [82] investigated the influence of ozone gas in reduction of the T-2 toxin contamination in malting wheat grains. When the processing time reached 130 min and the ozone concentration was 20 mg/L, the degradation rate of T-2 in malting grain extended up to 65%.

Probably the most efficient approach to reduce mycotoxins concentration is the use of adsorption powders. Olopade et al. [75] used differently modified montmorillonite clays as an adsorption agent mixed with T-2 contaminated maize, showing even six times T-2 concentration reduction within 4 weeks of storage at 30 °C. Similar results were achieved by Carson et. al. [83] with bentonite, but to efficiently reduce the T-2 toxin level, much more than 10 g/kg of bentonite must be used. New adsorbents based on tri-octahedral bentonites are very promising, absorbing a variety of mycotoxins reaching even 75% absorption rate versus ochratoxin A, but these have not yet been tested against T-2 toxin [84].

### 5.3. Biological Methods

The effect of treatment with lactic acid bacteria (LAB) on various mycotoxins contained in malting wheat grains was studied [85]. Analyses revealed that the presence of *Pediococcus pentosaceus* strains resulted in the greatest reduction of T-2. The concentration of T-2 toxin in malting wheat was decreased by 58%. The inhibition of T-2 and other mycotoxins probably resulted from toxins binding with LAB or from the detoxification of toxin with LAB. Nathanail et al. [76] showed that *Saccharomyces pastorianus* A15 lager yeast were able to reduce the mycotoxins levels in wort or to mitigate their effects by physical binding and/or conjugation. T-2 toxin reduction up to 31% was observed during the 96 h fermentation time. In different study [86], the detoxification properties of probiotic *Lactobacillus* sp. bacteria and *Saccharomyces cerevisiae* yeast toward various mycotoxins were investigated. After 24 h of incubation in the presence of *Lactobacillus* sp. strains, T-2 toxin concentration was reduced by 61% in relation to the initial quantity of toxin. Similarly, the concentration of the toxin after 24 h of incubation with *S. cerevisiae* strains was reduced by 61% of the initial concentration. Hassan and colleagues demonstrated that Bacillus spp. has significant T-2 toxin biodegrading capacity, leading to an 88% decontamination rate in substrates artificially contaminated with mycotoxin [87].

## 6. Conclusions

T-2 toxin is found in various regions worldwide and has adverse effects on both human and animal health. T-2 affects many organs and systems and exhibits the following characteristics: immunotoxicity, neurotoxicity, hepatotoxicity, nephrotoxicity, dermal toxicity, as well as disruption of the reproductive system. Currently, there is no specific therapy for T-2 toxin intoxication. Supportive measures such as superactivated charcoal administration if toxin was ingested is suggested. Different decontamination strategies have been found to mitigate the presence of T-2 toxin in agricultural products. There are physical, chemical, and biological methods, and depending on the technique used, the concentration of the toxin can be reduced by up to 90%. However, it may not be possible to completely prevent the formation of T-2 in agricultural commodities. Therefore, it is important to constantly monitor the level of contamination. Control and good agricultural practices in the pre-harvest, during, and post-harvest stages can significantly reduce T-2 contamination of agricultural commodities and cereal-based products. In the future, new or improved techniques of decontamination and degradation should be used to mitigate the concentration of T-2 in various agricultural commodities.

## Figures and Tables

**Table 1:** Substitution pattern of EPT of common trichothecenes (TCT).

Toxin	Type	R_1_	R_2_	R_3_	R_4_	R_5_
T-2	A	OH	OCOCH_3_	OCOCH_3_	H	OCOCH_2_CH(CH_3_)_2_
HT-2	A	OH	OH	OCOCH_3_	H	OCOCH_2_CH(CH_3_)_2_
Neosolaniol (NEO)	A	OH	OCOCH_3_	OCOCH_3_	H	OH
Diacetoxyscirpenol (DAS)	A	OH	OCOCH_3_	OCOCH_3_	H	H
Nivalenol (NIV)	B	OH	OH	OH	OH	=O
Deoxynivalenol (DON)	B	OH	H	OH	OH	=O
3-Acetyldeoxynivalenol (3-ADON)	B	OCOCH_3_	H	OH	OH	=O
15-Acetyldeoxynivalenol (15-AcDON)	B	OH	H	OCOCH_3_	OH	=O
Crotocin	C	H	OCOCH-CHCH_3_	H	Epoxide	
Roridin E	D	H	Macrocyclic ring		H	H
Verrucarin A	D	H	Macrocyclic ring		H	H

**Table 2:** Summary of T-2 toxin metabolites in in vivo study in chickens.

Number of Metabolite	Metabolite	Metabolic Pathway
1	HT-2 toxin (HT-2)	Hydrolysis
2	Neosolaniol (NEO)	
3	4-deacetylneosolaniol (4-deAc-NEO)	
4	3′-hydroxy-T-2 (3′-OH-T-2)	Hydroxylation
5	3′-hydroxy-HT-2 (3′-OH-HT-2)	
6	3′-Hydroxy-T-2-3-sulfate (3′-OH-T-2 3-SO3H)	Sulfonation
7	3′-Hydroxy-HT-2-3-sulfate (3′-OH-HT-2 3-SO3H)	
8	4′-Hydroxy-HT-2 (4′-OH-HT-2)	Hydroxylation
9	4′-OH-HT-2 isomer	
10	4′-Carboxyl-T-2 (4′-COOH-T-2)	Carboxylation
11	4′-COOH-T-2 isomer	
12	4′-Carboxyl-HT-2 (4′-COOH-HT-2)	
13	4′-COOH-HT-2 isomer	
14	4′-Carboxyl-3′-hydroxy-T-2 (4′-COOH-3′-OH-T-2)	
15	4′-COOH-3′-OH-T-2 isomer	
16	3′,4′-Dihydroxy-T-2 (3′,4′-di-OH-T-2)	Hydroxylation
17	3′,4′-di-OH-T-2 isomer	
18	4′,4′-Dihydroxy-T-2 (4′,4′-di-OH-T-2)	

## Data Availability

Not applicable.

## References

[1] Chen P., Xiang B., Shi H., Yu P., Song Y., Li S. (2020). Recent advances on type A trichothecenes in food and feed: Analysis, prevalence, toxicity, and decontamination techniques. Food Control.

[2] Milićević D.R., Škrinjar M., Baltić T. (2010). Real and Perceived Risks for Mycotoxin Contamination in Foods and Feeds: Challenges for Food Safety Control. Toxins.

[3] McCormick S.P., Stanley A.M., Stover N.A., Alexander N.J. (2011). Trichothecenes: From simple to complex mycotoxins. Toxins.

[4] Foroud N.A., Baines D., Gagkaeva T.Y., Thakor N., Badea A., Steiner B., Bürstmayr M., Bürstmayr H. (2019). Trichothecenes in Cereal Grains—An Update. Toxins.

[5] Arunachalam C., Doohan F.M. (2013). Trichothecene toxicity in eukaryotes: Cellular and molecular mechanisms in plants and animals. Toxicol. Lett..

[6] Nielsen C., Casteel M., Didier A., Dietrich R., Märtlbauer E. (2009). Trichothecene-induced cytotoxicity on human cell lines. Mycotoxin Res..

[7] Polak-Śliwińska M., Paszczyk B. (2021). Trichothecenes in Food and Feed, Relevance to Human and Animal Health and Methods of Detection: A Systematic Review. Molecules.

[8] Cardoza R.E., Malmierca M.G., Hermosa M.R., Alexander N.J., McCormick S.P., Proctor R.H., Tijerino A.M., Rumbero A., Monte E., Gutiérrez S. (2011). Identification of Loci and Functional Characterization of Trichothecene Biosynthesis Genes in Filamentous Fungi of the Genus *Trichoderma*. Appl. Environ. Microbiol..

[9] Proctor R.H., McCormick S.P., Kim H.-S., Cardoza R.E., Stanley A.M., Lindo L., Kelly A., Brown D.W., Lee T., Vaughan M.M. (2018). Evolution of structural diversity of trichothecenes, a family of toxins produced by plant pathogenic and entomopathogenic fungi. PLoS Pathog..

[10] Foroud N.A., Shank R.A., Kiss D., Eudes F., Hazendonk P. (2016). Solvent and Water Mediated Structural Variations in Deoxynivalenol and Their Potential Implications on the Disruption of Ribosomal Function. Front. Microbiol..

[11] Garvey G.S., McCormick S.P., Rayment I. (2008). Structural and Functional Characterization of the TRI101 Trichothecene 3-O-Acetyltransferase from *Fusarium sporotrichioides* and *Fusarium graminearum*: *Kinetic insights to combating fusarium head blight*. J. Biol. Chem..

[12] Pestka J.J. (2007). Deoxynivalenol: Toxicity, mechanisms and animal health risks. Anim. Feed. Sci. Technol..

[13] Wan Q., Wu G., He Q., Tang H., Wang Y. (2015). The toxicity of acute exposure to T-2 toxin evaluated by the metabonomics technique. Mol. BioSystems.

[14] Nayakwadi S., Ramu R., Kumar Sharma A., Kumar Gupta V., Rajukumar K., Kumar V., Shirahatti P.S., Rashmi L., Basalingappa K.M. (2020). Toxicopathological studies on the effects of T-2 mycotoxin and their interaction in juvenile goats. PLoS ONE.

[15] Pinton P., Oswald I.P. (2014). Effect of deoxynivalenol and other Type B trichothecenes on the intestine: A review. Toxins.

[16] Lancova K., Hajslova J., Poustka J., Krplova A., Zachariasova M., Dostalek P., Sachambula L. (2008). Transfer of Fusarium mycotoxins and ‘masked’ deoxynivalenol (deoxynivalenol-3-glucoside) from field barley through malt to beer. Food Addit. Contam. Part A.

[17] Male D., Wu W., Mitchell N.J., Bursian S., Pestka J.J., Wu F. (2016). Modeling the emetic potencies of food-borne trichothecenes by benchmark dose methodology. Food Chem. Toxicol..

[18] Pascari X., Maul R., Kemmlein S., Marin S., Sanchis V. (2020). The fate of several trichothecenes and zearalenone during roasting and enzymatic treatment of cereal flour applied in cereal-based infant food production. Food Control.

[19] Cope R.B., Gupta R.C. (2018). Chapter 75—Trichothecenes. Veterinary Toxicology.

[20] Rameshkumar G., Sikha M., Ponlakshmi M., Selva pandiyan A., Lalitha P. (2019). A rare case of Myrothecium species causing mycotic keratitis: Diagnosis and management. Med. Mycol. Case Rep..

[21] He J., Zhou T., Young J.C., Boland G.J., Scott P.M. (2010). Chemical and biological transformations for detoxification of trichothecene mycotoxins in human and animal food chains: A review. Trends Food Sci. Technol..

[22] Meneely J.P., Ricci F., van Egmond H.P., Elliott C.T. (2011). Current methods of analysis for the determination of trichothecene mycotoxins in food. TrAC Trends Anal. Chem..

[23] Karlovsky P., Suman M., Berthiller F., De Meester J., Eisenbrand G., Perrin I., Oswald I.P., Speijers G., Chiodini A., Recker T. (2016). Impact of food processing and detoxification treatments on mycotoxin contamination. Mycotoxin Res..

[24] Zhang J., You L., Wu W., Wang X., Chrienova Z., Nepovimova E., Wu Q., Kuca K. (2020). The neurotoxicity of trichothecenes T-2 toxin and deoxynivalenol (DON): Current status and future perspectives. Food Chem. Toxicol..

[25] Arcella D., Gergelova P., Innocenti M.L., Steinkellner H., European Food Safety Authority (EFSA) (2017). Human and animal dietary exposure to T-2 and HT-2 toxin. EFSA J..

[26] Zouagui Z., Asrar M., Lakhdissi H., Abdennebi E.H. (2017). Prevention of mycotoxin effects in dairy cows by adding an anti-mycotoxin product in feed. J. Mater. Environ. Sci..

[27] Cano-Sancho G., Valle-Algarra F.M., Jiménez M., Burdaspal P., Legarda T.M., Ramos A.J., Sanchis V., Marín S. (2011). Presence of trichothecenes and co-occurrence in cereal-based food from Catalonia (Spain). Food Control.

[28] Rosa Seus Arraché E., Fontes M.R.V., Garda Buffon J., Badiale-Furlong E. (2018). Trichothecenes in wheat: Methodology, occurrence and human exposure risk. J. Cereal Sci..

[29] Li Y., Wang Z., Beier R.C., Shen J., Smet D.D., De Saeger S., Zhang S. (2011). T-2 Toxin, a Trichothecene Mycotoxin: Review of Toxicity, Metabolism, and Analytical Methods. J. Agric. Food Chem..

[30] Moss M.O. (2002). Mycotoxin review–2. Fusarium. Mycologist.

[31] Nazari L., Pattori E., Terzi V., Morcia C., Rossi V. (2014). Influence of temperature on infection, growth, and mycotoxin production by Fusarium langsethiae and F. sporotrichioides in durum wheat. Food Microbiol..

[32] Nathanail A.V., Varga E., Meng-Reiterer J., Bueschl C., Michlmayr H., Malachova A., Fruhmann P., Jestoi M., Peltonen K., Adam G. (2015). Metabolism of the Fusarium Mycotoxins T-2 Toxin and HT-2 Toxin in Wheat. J. Agric. Food Chem..

[33] Edwards S.G., Imathiu S.M., Ray R.V., Back M., Hare M.C. (2012). Molecular studies to identify the Fusarium species responsible for HT-2 and T-2 mycotoxins in UK oats. Int. J. Food Microbiol..

[34] Lippolis V., Pascale M., Maragos C.M., Visconti A. (2008). Improvement of detection sensitivity of T-2 and HT-2 toxins using different fluorescent labeling reagents by high-performance liquid chromatography. Talanta.

[35] Kiš M., Vulić A., Kudumija N., Šarkanj B., Jaki Tkalec V., Aladić K., Škrivanko M., Furmeg S., Pleadin J. (2021). A Two-Year Occurrence of Fusarium T-2 and HT-2 Toxin in Croatian Cereals Relative of the Regional Weather. Toxins.

[36] Medina A., Magan N. (2011). Temperature and water activity effects on production of T-2 and HT-2 by Fusarium langsethiae strains from north European countries. Food Microbiol..

[37] Garai E., Risa A., Varga E., Cserháti M., Kriszt B., Urbányi B., Csenki Z. (2020). Qualifying the T-2 Toxin-Degrading Properties of Seven Microbes with Zebrafish Embryo Microinjection Method. Toxins.

[38] Makowska K., Obremski K., Gonkowski S. (2018). The Impact of T-2 Toxin on Vasoactive Intestinal Polypeptide-Like Immunoreactive (VIP-LI) Nerve Structures in the Wall of the Porcine Stomach and Duodenum. Toxins.

[39] Königs M., Mulac D., Schwerdt G., Gekle M., Humpf H.-U. (2009). Metabolism and cytotoxic effects of T-2 toxin and its metabolites on human cells in primary culture. Toxicology.

[40] Yagen B., Joffe A.Z. (1976). Screeing of toxic isolates of Fusarium poae and Fusarium sporotrichiodes involved in causing alimentary toxic aleukia. Appl. Environ. Microbiol..

[41] Sokolović M., Garaj-Vrhovac V., Šimpraga B. (2008). T-2 toxin: Incidence and toxicity in poultry. Arhiv za Higijenu Rada i Toksikologiju.

[42] Wannemacher R.W., Wiener S.L., Sidell F.R., Takafuji E.T., Franz D.R. (1997). Trichothecene mycotoxins. Med. Asp. Chem. Biol. Warf..

[43] Yang S., Li Y., Cao X., Hu D., Wang Z., Wang Y., Shen J., Zhang S. (2013). Metabolic Pathways of T-2 Toxin in in Vivo and in Vitro Systems of Wistar Rats. J. Agric. Food Chem..

[44] Kuca K., Dohnal V., Jezkova A., Jun D. (2008). Metabolic pathways of T-2 toxin. Curr. Drug Metab..

[45] Mackei M., Orbán K., Molnár A., Pál L., Dublecz K., Husvéth F., Neogrády Z., Mátis G. (2020). Cellular Effects of T-2 Toxin on Primary Hepatic Cell Culture Models of Chickens. Toxins.

[46] EFSA Panel on Contaminants in the Food Chain (CONTAM) (2011). Scientific Opinion on the risks for animal and public health related to the presence of T-2 and HT-2 toxin in food and feed. EFSA J..

[47] Yang S., De Boevre M., Zhang H., De Ruyck K., Sun F., Zhang J., Jin Y., Li Y., Wang Z., Zhang S. (2017). Metabolism of T-2 Toxin in Farm Animals and Human In Vitro and in Chickens In Vivo Using Ultra High-Performance Liquid Chromatography- Quadrupole/Time-of-Flight Hybrid Mass Spectrometry Along with Online Hydrogen/Deuterium Exchange Technique. J. Agric. Food Chem..

[48] Escrivá L., Font G., Manyes L. (2015). In vivo toxicity studies of fusarium mycotoxins in the last decade: A review. Food Chem. Toxicol..

[49] Rai R.B., Rahman S., Dixit H., Rai S., Singh B., Kumar H., Damodaran T., Dhama K. (2011). Analysis of feed ingredients for Afla and T-2 mycotoxins by ELISA in rural areas of Uttar Pradesh. Indian J. Vet. Pathol..

[50] Adhikari M., Negi B., Kaushik N., Adhikari A., Al-Khedhairy A.A., Kaushik N.K., Choi E.H. (2017). T-2 mycotoxin: Toxicological effects and decontamination strategies. Oncotarget.

[51] Sudakin D.L. (2003). Trichothecenes in the environment: Relevance to human health. Toxicol. Lett..

[52] Ueno Y. (1984). Toxicological features of T-2 toxin and related trichothecenes. Fundam. Appl. Toxicol..

[53] Wu Q.-H., Wang X., Yang W., Nüssler A.K., Xiong L.-Y., Kuča K., Dohnal V., Zhang X.-J., Yuan Z.-H. (2014). Oxidative stress-mediated cytotoxicity and metabolism of T-2 toxin and deoxynivalenol in animals and humans: An update. Arch. Toxicol..

[54] Ihara T., Sugamata M., Sekijima M., Okumura H., Yoshino N., Ueno Y. (1997). Apoptotic cellular damage in mice after T-2 toxin-induced acute toxicosis. Nat. Toxins.

[55] Yin H., Han S., Chen Y., Wang Y., Li D., Zhu Q. (2020). T-2 Toxin Induces Oxidative Stress, Apoptosis and Cytoprotective Autophagy in Chicken Hepatocytes. Toxins.

[56] Shinozuka J., Miwa S., Fujimura H., Toriumi W., Doi K. (2006). Hepatotoxicity of T-2 toxin, trichothecene mycotoxin. Mycotoxins.

[57] Rahman S., Sharma A.K., Singh N.D., Prawez S. (2020). Immunopathological effects of experimental T-2 mycotoxicosis in Wistar rats. Hum. Exp. Toxicol..

[58] Minervini F., Fornelli F., Lucivero G., Romano C., Visconti A. (2005). T-2 toxin immunotoxicity on human B and T lymphoid cell lines. Toxicology.

[59] Hymery N., Léon K., Carpentier F.G., Jung J.L., Parent-Massin D. (2009). T-2 toxin inhibits the differentiation of human monocytes into dendritic cells and macrophages. Toxicol. In Vitro.

[60] Seeboth J., Solinhac R., Oswald I.P., Guzylack-Piriou L. (2012). The fungal T-2 toxin alters the activation of primary macrophages induced by TLR-agonists resulting in a decrease of the inflammatory response in the pig. Vet. Res..

[61] Agrawal M., Bhaskar A.S.B., Rao P.V.L. (2015). Involvement of Mitogen-Activated Protein Kinase Pathway in T-2 Toxin-Induced Cell Cycle Alteration and Apoptosis in Human Neuroblastoma Cells. Mol. Neurobiol..

[62] Zhang X., Wang Y., Velkov T., Tang S., Dai C. (2018). T-2 toxin-induced toxicity in neuroblastoma-2a cells involves the generation of reactive oxygen, mitochondrial dysfunction and inhibition of Nrf2/HO-1 pathway. Food Chem. Toxicol..

[63] Weidner M., Lenczyk M., Schwerdt G., Gekle M., Humpf H.-U. (2013). Neurotoxic Potential and Cellular Uptake of T-2 Toxin in Human Astrocytes in Primary Culture. Chem. Res. Toxicol..

[64] Guo P., Liu A., Huang D., Wu Q., Fatima Z., Tao Y., Cheng G., Wang X., Yuan Z. (2018). Brain damage and neurological symptoms induced by T-2 toxin in rat brain. Toxicol. Lett..

[65] Gaigé S., Djelloul M., Tardivel C., Airault C., Félix B., Jean A., Lebrun B., Troadec J.-D., Dallaporta M. (2014). Modification of energy balance induced by the food contaminant T-2 toxin: A multimodal gut-to-brain connection. Brain Behav. Immun..

[66] Yang J.Y., Zhang Y.F., Liang A.M., Kong X.F., Li Y.X., Ma K.W., Jing A.H., Feng S.Y., Qiao X.L. (2009). Toxic effects of T-2 toxin on reproductive system in male mice. Toxicol. Ind. Health.

[67] Yang X., Zhang X., Zhang J., Ji Q., Huang W., Zhang X., Li Y. (2019). Spermatogenesis disorder caused by T-2 toxin is associated with germ cell apoptosis mediated by oxidative stress. Environ. Pollut..

[68] Shen J., Perveen A., Kaka N., Li Z., Dai P., Li C. (2019). Maternal Exposure to T-2 Toxin Induces Changes in Antioxidant System and Testosterone Synthesis in the Testes of Mice Offspring. Animals.

[69] Perveen A., Shen J., Ali Kaka N., Li C. (2020). Maternal Exposure to T-2 Toxin Affects Puberty Genes and Delays Estrus Cycle in Mice Offspring. Animals.

[70] Caloni F., Ranzenigo G., Cremonesi F., Spicer L.J. (2009). Effects of a trichothecene, T-2 toxin, on proliferation and steroid production by porcine granulosa cells. Toxicon.

[71] Hemmati A.A., Kalantari H., Jalali A., Rezai S., Zadeh H.H. (2012). Healing effect of quince seed mucilage on T-2 toxin-induced dermal toxicity in rabbit. Exp. Toxicol. Pathol..

[72] Pang V.F., Swanson S.P., Beasley V.R., Buck W.B., Haschek W.M. (1987). The toxicity of T-2 toxin in swine following topical application: I. Clinical signs, pathology, and residue concentrations. Fundam. Appl. Toxicol..

[73] Agrawal M., Yadav P., Lomash V., Bhaskar A.S.B., Lakshmana Rao P.V. (2012). T-2 toxin induced skin inflammation and cutaneous injury in mice. Toxicology.

[74] Shi H., Li S., Bai Y., Louzada Prates L., Lei Y., Yu P. (2018). Mycotoxin contamination of food and feed in China: Occurrence, detection techniques, toxicological effects and advances in mitigation technologies. Food Control.

[75] Olopade B.K., Oranusi S.U., Nwinyi O.C., Lawal I.A., Gbashi S., Njobeh P.B. (2019). Decontamination of T-2 Toxin in Maize by Modified Montmorillonite Clay. Toxins.

[76] Nathanail A.V., Gibson B., Han L., Peltonen K., Ollilainen V., Jestoi M., Laitila A. (2016). The lager yeast Saccharomyces pastorianus removes and transforms Fusarium trichothecene mycotoxins during fermentation of brewer’s wort. Food Chem..

[77] De Angelis E., Monaci L., Pascale M., Visconti A. (2012). Fate of deoxynivalenol, T-2 and HT-2 toxins and their glucoside conjugates from flour to bread: An investigation by high-performance liquid chromatography high-resolution mass spectrometry. Food Addit. Contam. Part A Chem. Anal. Control Expo. Risk Assess..

[78] Kuchenbuch H., Becker S., Schulz M., Cramer B., Humpf H.-U. (2018). Thermal stability of T-2 and HT-2 toxins during biscuit- and crunchy muesli-making and roasting. Food Addit. Contam. Part A Chem. Anal. Control Expo. Risk Assess..

[79] Wei J.-T., Wu K.-T., Sun H., Khalil M.M., Dai J.-F., Liu Y., Liu Q., Zhang N.-Y., Qi D.-S., Sun L.-H. (2019). A Novel Modified Hydrated Sodium Calcium Aluminosilicate (HSCAS) Adsorbent Can Effectively Reduce T-2 Toxin-Induced Toxicity in Growth Performance, Nutrient Digestibility, Serum Biochemistry, and Small Intestinal Morphology in Chicks. Toxins.

[80] Čolović R., Puvača N., Cheli F., Avantaggiato G., Greco D., Đuragić O., Kos J., Pinotti L. (2019). Decontamination of Mycotoxin-Contaminated Feedstuffs and Compound Feed. Toxins.

[81] Luo Y., Liu X., Li J. (2018). Updating techniques on controlling mycotoxins—A review. Food Control.

[82] Reinholds I., Gražina J., Bartkiene E., Zadeike D., Bartkevics V., Lele V., Cernauskas D., Cizeikiene D. (2016). Evaluation of ozonation as a method for mycotoxins degradation in malting wheat grains. World Mycotoxin J..

[83] Carson M.S., Smith T.K. (1983). Role of bentonite in prevention of T-2 toxicosis in rats. J. Anim. Sci..

[84] Vila-Donat P., Marín S., Sanchis V., Ramos A.J. (2019). New mycotoxin adsorbents based on tri-octahedral bentonites for animal feed. Anim. Feed Sci. Technol..

[85] Juodeikiene G., Bartkiene E., Cernauskas D., Cizeikiene D., Zadeike D., Lele V., Bartkevics V. (2018). Antifungal activity of lactic acid bacteria and their application for Fusarium mycotoxin reduction in malting wheat grains. LWT.

[86] Chlebicz A., Śliżewska K. (2020). In Vitro Detoxification of Aflatoxin B(1), Deoxynivalenol, Fumonisins, T-2 Toxin and Zearalenone by Probiotic Bacteria from Genus *Lactobacillus* and *Saccharomyces cerevisiae* Yeast. Probiotics Antimicrob. Proteins.

[87] Hassan Z.U., Al Thani R., Alsafran M., Migheli Q., Jaoua S. (2021). Selection of *Bacillus* spp. with decontamination potential on multiple *Fusarium* mycotoxins. Food Control.

